# Amino Acids and Developmental Origins of Hypertension

**DOI:** 10.3390/nu12061763

**Published:** 2020-06-12

**Authors:** Chien-Ning Hsu, You-Lin Tain

**Affiliations:** 1 Department of Pharmacy, Kaohsiung Chang Gung Memorial Hospital, Kaohsiung 833, Taiwan; chien_ning_hsu@hotmail.com; 2 School of Pharmacy, Kaohsiung Medical University, Kaohsiung 807, Taiwan; 3 Department of Pediatrics, Kaohsiung Chang Gung Memorial Hospital and Chang Gung University College of Medicine, Kaohsiung 833, Taiwan; 4 Institute for Translational Research in Biomedicine, Kaohsiung Chang Gung Memorial Hospital and Chang Gung University College of Medicine, Kaohsiung 833, Taiwan

**Keywords:** amino acid, developmental origins of health and disease (DOHaD), gut microbiota, hypertension, nitric oxide, nutrient-sensing signal, oxidative stress, pregnancy

## Abstract

During pregnancy, amino acids are important biomolecules that play essential roles in fetal growth and development. Imbalanced amino acid intake during gestation may produce long-term morphological or functional changes in offspring, for example, developmental programming that increases the risk of developing hypertension in later life. Conversely, supplementation with specific amino acids could reverse the programming processes in early life, which may counteract the rising epidemic of hypertension. This review provides an overview of the evidence supporting the importance of amino acids during pregnancy and fetal development, the impact of amino acids on blood pressure regulation, insight from animal models in which amino acids were used to prevent hypertension of developmental origin, and interactions between amino acids and the common mechanisms underlying development programming of hypertension. A better understanding of the pathophysiological roles of specific amino acids and their interactions in developmental programming of hypertension is essential so that pregnant mothers are able to benefit from accurate amino acid supplementation during pregnancy in order to prevent hypertension development in their children.

## 1. Introduction

Current evidence indicates that the origins of hypertension can be found in early life [1,2,3,4]. Nutrition is the major intrauterine environmental factor that alters fetal morphology and function through a process termed fetal programming [5]. An imbalance in this process may cause hypertension in later life [6]. This notion has become globally recognized as the developmental origins of health and disease (DOHaD) concept [7]. Conversely, growing evidence suggests that the use of intervention strategies in the early phases of developmental plasticity can ameliorate or reverse the adverse effects associated with developmental programming through reprogramming [8]. Recent research studies have started paying more attention to the use of nutritional interventions as reprogramming strategies to prevent hypertension of developmental origin [6,9].

**Figure 1:**
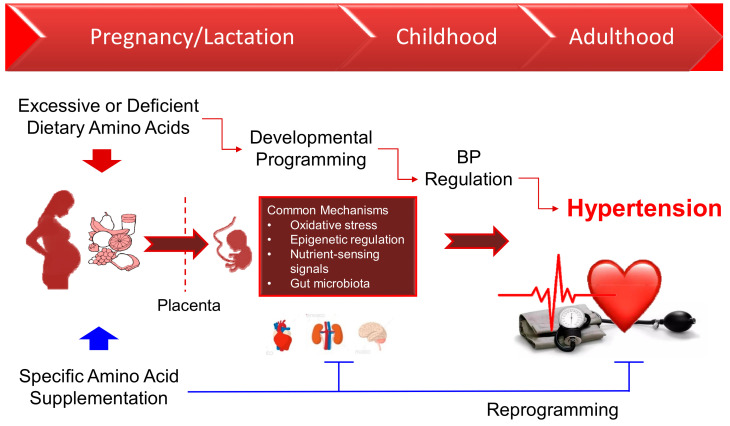
Schematic illustration of the association between amino acid intake in pregnancy, developmental programming, and increased vulnerability to hypertension in adult offspring. The solid red arrow line indicates that an oversupply or deficiency of amino acids in pregnancy can lead to developmental programming of hypertension in adult offspring. There are several common mechanisms, including oxidative stress, epigenetic regulation, nutrient-sensing signals, and gut microbiota involved in this process. The solid blue line indicates the beneficial effects of amino acid supplementation on hypertension of developmental origin. BP, blood pressure.

Twenty amino acids that make up proteins are essential nutrients in a healthy diet that ensure optimal growth and maintenance in humans. During gestation, an exceptional stage of life defined by rapid fetal growth and development, adequate dietary amino acid availability is essential to ensure the development of healthy offspring [10,11]. Despite the fact that protein intake recommendations in pregnancy are provided as estimated average requirement (EAR) and recommended dietary allowances (RDA) values [12], there is a lack of specific amino acid recommendations for pregnant women [11]. Some amino acids and types of proteins have been associated with blood pressure (BP) [13,14,15]; however, the potential effects of maternal amino acid intake on progeny BP are largely unknown. This review, therefore, highlights evidence on the impact of amino acids during pregnancy on offspring hypertension, as well as the role of amino acid supplementation as a reprogramming strategy in the prevention of hypertension of developmental origin. The associations between amino acids in pregnancy and the risk of hypertension in adult offspring are illustrated in Figure 1.

Relevant peer-reviewed journal articles published in English were identified in the PubMed and MEDLINE databases (the last search was conducted on 25 March 2020); different combinations of the following search terms were used: “amino acids,” “hypertension,” “blood pressure,” “developmental programming,” “DOHaD,” “offspring,” “progeny,” “pregnancy,” “mother,” “maternal,” and “perinatal.” Bibliographic references from eligible articles were reviewed, and any additional studies were selected.

## 2. Amino Acid Requirements during Pregnancy and Fetal Development

### 2.1. Amino Acid Requirements in Pregnancy

Pregnancy is associated with hypoaminoacidemia during fasting, which is evident early in gestation and persists throughout pregnancy [16,17]. In particular, there is a more profound reduction in glucogenic amino acids—alanine, serine, threonine, glutamine, and glutamate [16]. The current RDA of the protein Dietary Reference Intake (DRI) is 1.1 g/kg/day during pregnancy, which represents an increase from 0.8 g/kg/day in the non-pregnant state [12]. It is often stated that the level of amino acids needs to increase in proportion to the increased protein needs during pregnancy; however, very few studies have reported specific amino acid requirements in human pregnancy [11]. A previous study showed that lysine requirements during late gestation increase by 27% when compared to the requirements in early gestation [18]. Another report demonstrated that there is a 40% higher requirement for phenylalanine during late gestation than during early gestation in human pregnancy [19]. In swine models, the requirements for threonine have been shown to increase by 55%, lysine by 45%, isoleucine by 63%, and tryptophan by 35% during the late stages of pregnancy when compared to the early stages [11]. Since isoleucine, a branched chain amino acid (BCAA), is the major source of nitrogen for ureogenic amino acids [11], it is speculated that the adaptive increase is aimed at overall conservation of nitrogen and increased protein synthesis. However, whether other BCAA requirements are increased and the exact mechanism of this adaptation remain unknown. These observations suggest potential implications for gestation-stage-specific dietary amino acid recommendations.

### 2.2. Amino Acid Transport in the Placenta

**Figure 2:**
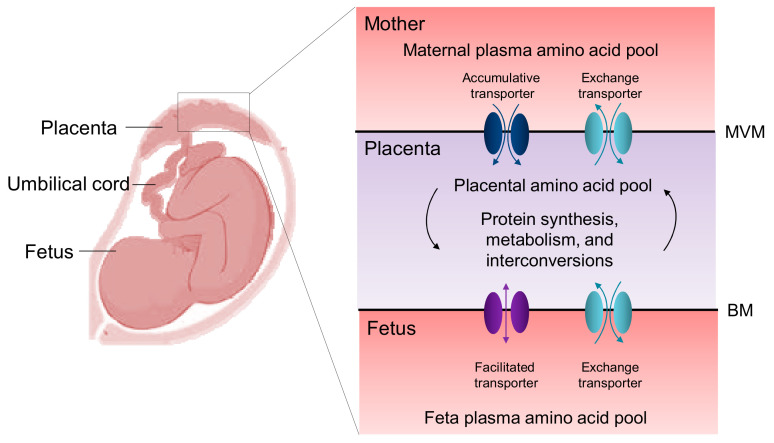
Schematic illustration of the placental amino acid transporters within the microvillous membrane (MVM) and basal membrane (BM) of the placental syncytiotrophoblast. Accumulative transporters located in the MVM mediate the uptake from the maternal circulation until their accumulative potential is reached. Exchange transporters mediate the net influx of abundant external amino acids in exchange for the efflux of relatively more abundant intracellular amino acids. Facilitated transporters on the BM mediate the efflux of amino acids down the concentration gradient into the fetal circulation. The placental amino acid pool can be regulated by protein synthesis, metabolism, and interconversions.

The fetal plasma concentrations of most amino acids are significantly higher than the maternal concentrations [20], indicating active amino acid transport across the placenta, from the maternal to the fetal circulation [21,22]. As shown in Figure 2, three functional types of amino acid transport systems have been identified in the placenta: accumulative, exchange, and facilitated transporters [23].

All of them belong to the solute carrier (SLC) superfamily. The accumulative transporters mediate the net uptake of specific amino acids across the maternal-facing microvillous membrane (MVM) into the syncytiotrophoblast, leading to the creation of concentration gradients to drive the uptake of other extracellular amino acids via amino acid exchange transporters. These exchange transporters on the MVM switch intracellular amino acids for other exchange transporter-specific amino acids in the maternal plasma. On the basal membrane (BM) of the syncytiotrophoblast, the facilitated transporters facilitate the net efflux of specific amino acids across the BM into the fetal circulation down their concentration gradients [23]. Table 1 provides a summary of the amino acid transport systems identified in the human placenta [24,25,26,27,28,29,30,31,32,33].

Increased expression/activity of SLC7A5 [34] and SLC7A8 [34] and decreased expression/activity of SLC7A1 [35] in the kidneys have been reported to be relevant to hypertension. However, the role of placental amino acid transporters in hypertension of developmental origin has not been adequately studied.

Of note is that placental protein synthesis, metabolism, and interconversions can together influence the pool of amino acids available for transport [36]. Additionally, the regulation of placental amino acid transfer is mainly mediated by nutrient-sensing signaling, such as the mechanistic target of rapamycin (mTOR) pathway [36]. mTOR forms two multiprotein complexes, mTOR complex 1 (mTORC1) and 2 (mTORC2) [37,38]. mTORC1, which consists of mTOR, mammalian lethal with SEC13 protein 8 (GβL), Raptor, and domain-containing mTOR-interacting protein (DEPTOR), is inhibited by rapamycin; it unifies multiple signals that promote cellular growth and catabolic processes during stress [37]. mTORC2, which consists of mTOR, Rictor, GβL, Sin1, PRR5/Protor-1, and DEPTOR, promotes cell survival through the activation of Akt [38]. mTOR signaling regulates the activity of several key placental amino acid transporters. Previous studies have reported that placental mTOR activity, amino acid transfer, and amino acid transporter activity are decreased in intrauterine growth retardation (IUGR) [39,40,41]. Additionally, inhibition of mTOR by rapamycin significantly reduces the activity of system A, system L, and taurine amino acid transporters [41]. In short, amino acid concentrations in the fetal circulation are tightly controlled by the placenta, which is decisive for normal fetal development and lifelong health effects.

### 2.3. Amino Acids and Fetal Development

As we reviewed elsewhere [6], excessive or insufficient consumption of a specific nutrient, such as protein, has been linked to adverse fetal outcomes. One famous example is the Dutch famine study, which demonstrated that undernutrition in pregnancy is related to an increased risk of developing IUGR and subsequent hypertension in adult offspring [42]. There is also an association between high-protein intake in pregnancy and the risk of high BP in adult offspring [43,44]. Yet, few epidemiological human studies have investigated the impact of excessive or insufficient intake of a particular amino acid on fetal development and on the offspring outcomes. The total amino acid concentrations have been shown to be related to fetal outcome, particularly, infant birth weight. A previous study showed positive correlations among the concentrations of arginine, ornithine, serine, lysine, proline, and neonatal birth weight [45]. Arginine is a common substrate for nitric oxide (NO) and polyamines (putrescine, spermine, and spermidine), both of which are crucial for fetal development and placental angiogenesis [46]. On the other hand, serine is not transported to the fetus in any significant quantity [47]. Thus, these correlations do not necessarily suggest that these amino acids play key roles in fetal growth. As the changes in a particular amino acid may affect the metabolic processes of other amino acids, additional studies have focused not only on individual amino acids but also on the balance of the amino acid pool in fetal development, and further studies are urgently warranted.

## 3. Amino Acids and Hypertension

### 3.1. The Role of Amino Acids in the Regulation of BP

Currently, approximately 500 naturally occurring amino acids are known [48]. Among them, more than a few amino acids have been linked to BP regulation. BP is tightly regulated by several organs, such as the brain, kidneys, and blood vessels. In conscious rats, intracisternal injections of the amino acids proline, arginine, cysteine, glutamate, aspartic acid, and asparagine produce pressor responses, while serine, alanine, taurine, and glycine produce depressor responses [49]. In blood vessels, arginine, homoarginine, and tryptophan are known to exert regulatory effects on the development of atherosclerosis [50]. Arginine is a substrate for NO, which plays a key role in endothelium-dependent vasodilatation in blood vessels [51]. Additionally, several arginine-related amino acids are involved in BP control. Citrulline can be used in the kidneys to produce arginine de novo. In spontaneously hypertensive rats, citrulline supplementation can increase renal NO production and prevent hypertension [52]. Protein arginine methylation results in the production of asymmetric and symmetric dimethylarginine (ADMA and SDMA), both of which cause vasoconstriction via NO inhibition [53,54]. Homoarginine is a nonproteinogenic amino acid that is structurally closely related to arginine. Like arginine, homoarginine has been reported to be a substrate in the synthesis of NO [55]. Methionine is an essential amino acid; among its metabolic byproducts, homocysteine, when elevated, may induce ADMA production, impair endothelial function, and increase BP [56]. Another sulfur-containing amino acid, cysteine, is the substrate for hydrogen sulfide (H_2_S). In addition, cysteine is a component of glutathione, an important antioxidant molecule in our body. As both glutathione and H_2_S signaling are closely linked to BP regulation [57,58], cysteine is considered to have an antihypertensive effect [59]. Likewise, taurine is a sulfur-containing amino acid with a vasodilator effect [60]. As reviewed elsewhere [61], the antihypertensive effect of taurine supplementation has been investigated in various hypertensive rat models. Furthermore, tryptophan and its metabolites have been shown to induce vasodilatation in a dose-dependent manner in the blood vessels [62]. These observations indicate that certain amino acids exhibit organ- or tissue-specific effects on the regulation of BP.

### 3.2. Dietary Amino Acids and Established Hypertension

There is some epidemiological evidence of a connection between dietary amino acids and hypertension. Several amino acids, as mentioned above, interfere with BP regulation; among them, dietary arginine supplementation has shown beneficial effects by lowering both systolic and diastolic BP in patients with hypertension [63,64]. However, in studies focusing on a usual diet, excluding supraphysiological intake through dietary supplementation, dietary arginine was not found to be associated with BP [65,66]. A meta-analysis study recruiting a total of 139 adults from five trials demonstrated that citrulline supplementation has no beneficial effect on BP [67]. Dietary alanine was shown to have a positive relation with BP in the INTERMAP study and in the THIS-DIET study [68,69]. Additionally, the associations of three aromatic amino acids—phenylalanine, tyrosine, and tryptophan—with the risk of hypertension were examined [70]. In this cohort study, which implemented a three-year follow-up assessment, a positive relationship was observed between a high intake of phenylalanine, but not tyrosine or tryptophan, and hypertension [71]. Another study showed that plasma phenylalanine, together with branched chain amino acids (BCAAs), has a positive association with both systolic and diastolic BP [72]. Regarding BCAAs, the results are conflicting: dietary BCAAs have been shown to be not associated [73], positively associated [70], or even negatively associated [74], with the risk of hypertension. Probably because of the varying study populations used and the differences in the study design and end points, there was little consistency among the BCAAs reported to be associated with the risk of hypertension. Furthermore, the standardization of instrumentation used in large research populations will open new horizons for scientists seeking to understand the impact of BCAAs on hypertension. Moreover, a high dietary intake of glutamate has been reported to be associated with low systolic and diastolic BP [68], while this finding was not supported by another study [71]. Likewise, the association between homocysteine and BP remains inconclusive [56]. A meta-analysis of seven trials showed that taurine supplementation at doses ranging from 1 to 6 g/day for one day to 12 weeks resulted in a mean reduction of ~3 mmHg in both systolic and diastolic BP [75]. Another meta-analysis of eight observational studies showed an inverse association between the consumption of dietary plant proteins and hypertension [76]. Plant proteins have a reduced content of some essential amino acids, such as methionine, lysine, and tryptophan, in comparison with animal proteins [77]. On the other hand, vegetarians have a significantly higher intake of the non-essential amino acids arginine, glycine, alanine, and serine [77]. Vegetarians are also more likely to be exposed to a low content of other putative metabolic stressors, such as saturated fats and certain lipid-derived compounds present in protein sources of animal origin. Although evidence suggests a beneficial effect of plant proteins on BP [76,77,78,79,80], more data are needed to show which specific amino acids from plant proteins relate to BP. Given the large variability in methodologies used for assessing amino acid levels, the complexity of amino acid interactions, and the heterogeneity in the study populations recruited, it is not possible to draw robust conclusions on the effects of certain amino acid intakes on BP in humans [14].

Since epidemiological studies do not dissect the physiological and molecular mechanisms by which hypertension is created, animal models allowing full control over dietary manipulations are essential in the discovery of the mechanisms that drive the programming processes and the development of specific amino acids as reprogramming interventions before clinical translation to human application.

## 4. Insight from Animal Models Targeting Amino Acids to Prevent Hypertension of Developmental Origin

Several animal models related to amino acid intake in pregnancy have been conducted to induce hypertension in offspring. As we reviewed elsewhere [6], models of low protein feeding in rodents, ranging from 6–9%, induce a rise in BP in adult offspring. These studies demonstrate that more severe protein restrictions tend to lead to the earlier development of hypertension [79,80,81,82,83]. Similarly, a maternal low protein diet has been reported to program hypertension-related disorders in adult offspring in other species, such as pigs, sheep, and cows [84,85].

On the other hand, oversupply or deficiency of specific amino acids in pregnancy have also been used to explore the mechanisms of developmental programming of hypertension [6]. We recently found that feeding pregnant rats with a high-methyl-donor diet or a methyl-deficient diet resulted in programmed hypertension in their male adult offspring [86]. Such methyl-donor nutrients include methionine, choline, folic acid, and vitamins B2, B6, and B12. Since these methyl-donor nutrients are critical intermediates or cofactors for one-carbon metabolism [87], this leads to the notion that dysregulated one-carbon metabolism may be a critical determinant of the programming of hypertension.

Conversely, certain amino acids can be used as reprogramming interventions to reverse the early-life insults induced by programmed processes and can consequently protect offspring against adverse outcomes. In the current review, we only focus on amino acid supplementation starting during pregnancy as a reprogramming strategy to prevent hypertension of developmental origin in rodent animal models, as listed in Table 2 [52,88,89,90,91,92,93,94,95,96,97,98,99,100,101,102,103,104,105,106]. This list is by no means complete and is likely to grow quickly, as the study of DOHaD-related disorders is a flourishing field. Rats grow rapidly during their childhood and reach sexual maturity at around 5–6 weeks of age. In adulthood, one rat month is equivalent to three human years [107]. Female rats enter menopause between the ages of 15 and 20 months. Accordingly, Table 2 lists the timing of developing hypertension evaluated at different ages, which allows calculations to refer to humans of a specific age group.

### 4.1. Arginine

Using an oral range of 3–100 g/day, arginine supplementation has been studied in human diseases as a method to improve NO bioavailability [108]. Single doses exceeding 9 g and a dosing regimen of over 30 g/day have been reported to cause gastrointestinal upset [109]. Thus far, the benefits of arginine from human trials remain inconclusive [110]. As shown in Table 2, perinatal arginine supplementation combined with taurine and antioxidants protects adult offspring against hypertension in spontaneously hypertensive rats (SHRs) and Fawn-hooded hypertensive (FHH) rats, two commonly used genetic hypertensive rat models [88,89,90,91]. In SHR, the BP-lowering effect of combined perinatal arginine and taurine supplementation continues to 48 weeks of age [91], which is equivalent to human young adulthood. Although arginine supplementation alone during the post-weaning period can prevent hypertension in offspring rats exposed to maternal caloric restriction or diabetes [111,112], whether perinatal arginine supplementation alone is associated with these effects has not been elucidated. Of note is that differential gene expression in two-day-, two-week-, and 48-week-old rats varies between control SHRs and SHRs treated with combined arginine and taurine supplementation, but the treatment alters only a few genes toward the normotensive control Wistar Kyoto (WKY) phenotype. These findings suggest that the persistent antihypertensive effect of amino acid supplementation might be epigenetic and related to renal transcriptome changes [91]. Furthermore, supplementation of arginine during the gestational period has been shown to have protective effects on IUGR in ovine and swine [113,114]. However, currently, the reprogramming effects of arginine supplementation in pregnancy, other than in terms of IUGR, have not been fully examined in these species.

### 4.2. Taurine

Table 2 indicates that taurine is the most commonly supplemented amino acid in pregnancy for studying programmed hypertension. Taurine is the most abundant sulfur-containing amino acid [115] and is mainly acquired from dietary sources, despite the fact that it can be synthesized from cysteine. During pregnancy, taurine accumulates in maternal tissues and is released to the fetus via the placenta [116]. In the human body, the most critical period for taurine exposure is during perinatal life, as its content is the highest during early postnatal life and declines with advancing age [116]. In rats, dietary taurine supplementation has been reported to prevent hypertension induced by a high-salt, high-fructose diet as well as various genetic hypertensive models [60,61,117,118]. Taurine has several potentially beneficial antihypertensive effects that involve the regulation of NO and H_2_S, the renin–angiotensin system (RAS), oxidative stress, and sympathetic activity [60,61,119]. Table 2 shows that combined use of taurine and arginine in the perinatal period causes a reduction of BP in SHRs as well as in FHH rats [88,89,90,91]. Perinatal taurine supplementation alone has also been shown to prevent hypertension in SHRs and stroke-prone spontaneously hypertensive rats (SHRSP) [94,95]. Additionally, maternal taurine supplementation protects adult offspring against hypertension programmed by maternal high-sugar intake or diabetes [92,93]. However, the long-term reprogramming effects of perinatal taurine supplementation on an offspring’s BP in later life still requires further clarification.

### 4.3. Citrulline

Citrulline is a non-essential amino acid that is made naturally in the human body, found in food (e.g., watermelon), and is available as a dietary supplement [120]. As citrulline can bypass hepatic metabolism and can be converted to arginine in the renal system, oral citrulline supplementation has been considered as an add-on therapy to raise plasma arginine concentrations and to increase NO production [120]. In humans, citrulline supplementation as a single oral dose, ranging between 2 and 15 g, is safe and well tolerated [121]. Following oral citrulline supplementation, circulating arginine concentrations reach their peak after 1–2 h [121]. In pregnancy, citrulline undergoes limited degradation in the placenta, being efficiently transferred from the mother to the fetus in favor of fetal development [122]. Thus far, evidence suggests that there are beneficial effects of citrulline supplementation on cardiometabolic health [123]. However, the long-term effects of citrulline supplementation in pregnancy on offspring outcomes remain largely unknown.

Citrulline supplementation has been used in pregnancy and lactation as a reprogramming intervention to protect adult rat offspring against hypertension in several rat models, including maternal caloric restriction [96], prenatal dexamethasone exposure [97], streptozotocin-induced diabetes [98], and maternal N^G^-nitro–L-arginine methyl ester (L-NAME) exposure [99,100]. Additionally, perinatal citrulline supplementation can restore NO bioavailability to prevent the transition of prehypertension to hypertension in spontaneously hypertensive rats [52]. Of note is that a 50% caloric restriction in pregnant ewes significantly reduced the total concentrations of α-amino acids (particularly serine, arginine, and BCAAs) in maternal and fetal plasma at both mid- and late-gestation [124]. Similar to the protein restriction diet model [79,80,105], 50% caloric restriction was found to cause IUGR, a common adverse outcome of maternal undernutrition [42,124]. These findings suggest that caloric restriction in pregnancy is equivalent to undernutrition and occurs with certain amino acid deficiencies. In a maternal NO deficiency rat model, maternal citrulline supplementation prevented hypertension programmed by L-NAME exposure, which is associated with more than 300 genes, and exhibited a significant change in the renal transcriptome in adult offspring [100]. These findings suggest that early citrulline supplementation has a long-term impact on the renal transcriptome. Thus, the implications of epigenetic regulation by citrulline at an early stage of programming deserve further clarification.

### 4.4. Cysteine

Like taurine, cysteine is another sulfur-containing amino acid [62]. Cysteine is also known to be rate-limiting for the synthesis of glutathione [59]. Cysteine supplementation has been used to create endogenous H_2_S in experimental studies [58]. Although early post-weaning cystine supplementation has been reported to prevent hypertension in high-salt-treated SHRs [125], gestational supplementation with cysteine has not yet been examined in developmental models of hypertension. As cysteine tends to be absorbed into cells where it cannot exhibit its antioxidant property, N-acetylcysteine (NAC), a stable cysteine analogue, is often used instead for this purpose. As shown in Table 2, the antihypertensive effects of perinatal NAC therapy have been reported in several animal models, including prenatal dexamethasone treatment and a postnatal high-fat diet [101], suramin-induced pre-eclampsia [102], L-NAME exposure [103], and maternal nicotine exposure [104]. Notably, the reprogramming effect of perinatal NAC supplementation is persistent in offspring rats at 8 months of age and correlates with early stages of middle adulthood in humans [104].

### 4.5. Others

There are other reprogramming interventions related to amino acids by which hypertension could be prevented in adult offspring, such as supplementation with glycine [105] and BCAAs [106]. First, supplemental glycine might have potential benefits on human disorders, as it contributes to glutathione synthesis [126]. Only one study has shown that perinatal glycine supplementation protects offspring against hypertension programmed by a maternal low-protein intake [105]. Second, BCAA supplementation in pregnancy is able to prevent hypertension programmed by maternal caloric restriction in adult offspring [106]. In the human body, BCAAs not only act as building blocks for protein synthesis but also act as a fuel source and regulate autophagy via the activation of mTOR [127]. Given the fact that the few studies that have addressed the association of BCAAs with hypertension have been inconclusive [72,73,74,127], there remains a need to better understand the reprogramming effects for perinatal BCAA use, especially in hypertension.

## 5. Common Mechanisms in the Developmental Programming of Hypertension

As various manipulations of amino acid supply in pregnancy create very similar protective effects against hypertension in adult offspring, there might be some common mechanisms that contribute to their beneficial effects on programmed hypertension. To date, several mechanisms have been linked to the developmental programming of hypertension [128,129,130,131,132,133]. Some of the mechanisms that have been related to the beneficial effects of amino acids include oxidative stress, epigenetic regulation, nutrient-sensing signals, and gut microbiota (Figure 1). Here, each of these is discussed in turn.

### 5.1. Oxidative Stress

Oxidative stress reflects an imbalance between the production of reactive oxygen species and antioxidant defense. NO, a free radical, plays a role in oxidative stress, and NO deficiency and increased oxidative stress are involved in the pathogenesis of hypertension [134]. Exposure to early-life oxidative stress can increase the risk of developing hypertension in later life [135]. Diverse nutritional insults in pregnancy have been reported to induce programmed hypertension attributed to oxidative stress, including caloric restriction [96], a low-protein diet [81], a methyl-donor diet [87], a high-fat diet [136], and a high-fructose diet [137]. Although a maternal low-protein diet leads to a decrease in the total amino acid concentration in the fetal circulation [138], whether the consumption of a specific amino acid that is deficient in pregnancy may result in oxidative stress and programmed hypertension in adult offspring remains unclear.

Several amino acids have antioxidant properties. The major antioxidant nutrient in the human body is glutathione, which is a tripeptide comprising cysteine, glutamate, and glycine. Several studies have reported that supplementation with NAC, a stable analogue of cysteine, can reduce oxidative stress and protect offspring against hypertension [101,102,103,104]. Additionally, glycine supplementation protects offspring against hypertension programmed by maternal low-protein intake [105]. Thus, these findings suggest that the suppression of oxidative stress may contribute to the antihypertensive effects of amino acid supplementation. However, whether the antioxidative ability of these amino acids themselves is important for lowering the BP in concert with other BP-reducing actions requires further elucidation. The restoration of NO depletion in pregnancy also contributes to the protective mechanisms that underlie programmed hypertension [132]. Perinatal supplementation with certain amino acids, including arginine [89], citrulline [96,97,98,99,100], and NAC [102], can restore NO bioavailability and can protect adult offspring against the development of hypertension.

### 5.2. Epigenetic Regulation

Epigenetic regulation processes, such as DNA methylation, histone modifications, and noncoding RNAs, are involved in mediating the effects of early-life nutritional influences on lifelong health [130]. DNA methylation is dependent on the one-carbon metabolism pathway [139]. Several amino acids, such as glycine, histidine, methionine, and serine, are involved in this pathway as they supply methyl donors for DNA and protein synthesis [139].

A low-protein diet in pregnancy has been reported to influence promoter methylation status and the expression of the glucocorticoid receptor (GR) and peroxisome proliferator-activated receptor (PPAR) genes of mice offspring via the acetylation of histones H3 and H4 and the methylation of H3K4 [135]. Importantly, emerging evidence indicates that both the GR and PPARs play important roles in hypertension of developmental origin [140,141]. Additionally, a low-protein diet has been associated with DNA hypermethylation of the *agtr1b* gene, which is implicated in hypertension [142]. Since our previous study demonstrated that feeding pregnant rats with a high-methyl-donor diet or a methyl-deficient diet causes a rise in BP in their male adult offspring [87], whether these amino acids involved in the one-carbon metabolism pathway play roles in the DNA methylation of genes related to BP regulation deserves further study. Currently, only a few studies have reported, using high-throughput DNA sequencing technologies, that early amino acid supplementation permanently alters the transcriptome expression profile in offspring [129]. Since arginine/taurine [91] and citrulline [97] supplementation in early life can alter various genes that drive the programming processes that affect the lifelong health of offspring, a better understanding of the underlying epigenetic mechanisms is urgently required. Overall, these studies support the idea that amino acid supplementation in pregnancy can epigenetically program the development of hypertension in later life. Nevertheless, the detailed mechanisms underlying the epigenetic modulation of particular genes by different types of amino acids still require additional study.

### 5.3. Nutrient-Sensing Signals

Imbalanced nutrition and metabolic insults in early life can disturb nutrient-sensing signals that play key roles in fetal metabolism and development [131]. PPARs, mTOR, silent information regulator transcript (SIRT), PPARγ coactivator-1α (PGC-1α), and cyclic adenosine monophosphate (AMP)-activated protein kinase (AMPK) are well-known nutrient-sensing signals [143]. Activation of AMPK by an increased NAD^+^/NADH ratio or activation of SIRT1 by an increased mitochondrial AMP/adenosine triphosphate (ATP) ratio can affect PGC-1α activity, thereby promoting mitochondria biogenesis [144]. The interplay among SIRT1, AMPK, mTOR, and amino acids tightly regulates autophagy [145]. Certain amino acids can activate the amino acid sensors upstream of mTORC1 to inhibit autophagy [36]. Among the various amino acids, leucine, phenylalanine, and tyrosine are the most potent for inhibiting autophagy [146]. Likewise, BCAAs can regulate autophagy via the activation of mTOR [127]. Hypertension programmed by a maternal methyl-donor diet is associated with the reduced expression of nutrient-sensing signaling in several forms, including *Sirt1, Pparb, Pparg,* and *Prkaa2* [87]. Conversely, activation of the AMPK/SIRT1/PGC-1α pathway can reverse the programming process and prevent hypertension in adult offspring [147].

On the other hand, amino-acid-dependent signaling driven by maternal nutritional interventions has been found to regulate PPARs and their target genes, thereby generating the programming of hypertension [141,146]. Several PPAR target genes related to oxidative stress and the RAS, such as *Nos2*, *Nos3*, *Sod2*, *Nrf2*, *Sirt7*, *Ren*, and *Sgk1*, are implicated in hypertension. Furthermore, PPARγ can increase several sodium transporters to increase sodium reabsorption, leading to programmed hypertension [103]. To sum up, these findings closely link amino acids to nutrient-sensing signals and hypertension of developmental origin. It will be necessary to study the mechanisms that underlie the interactions between specific amino acids and nutrient-sensing signals and their impacts on the hypertension programming process.

### 5.4. Gut Microbiota

Maternal nutritional insults can impair the gut microbial balance, leading to consequent adverse offspring outcomes, including hypertension [148]. The gut microbiota produces a variety of metabolites that are detectable in the host circulation, including short-chain fatty acids, small organic acids, bile acids, vitamins, and choline metabolites [149]. Additionally, the gut microbiota can metabolize almost all essential amino acids, which is critical for amino acid metabolism [150]. The catabolism of amino acids plays a key role in regulating the intestinal barrier and immune response [151]. Some functional amino acids, like tryptophan, glutamine, methionine, and BCAAs, have been shown to have beneficial effects on the gut-associated immune system [152]. Accordingly, the gut microbiota not only alter the pool of amino acids transported from the intestine to the circulation, they also secrete various metabolites characterized by nitrogen- and sulfur-containing materials.

Several proposed mechanisms, such as increased sympathetic activity, activation of the RAS, alterations of microbial metabolite short-chain fatty acids and trimethylamine-N-oxide, inhibition of NO, and mediation of the H_2_S signaling pathway, link gut microbiota dysbiosis to hypertension [152]. On the other hand, probiotics are emerging as a functional food supplement that provides several health benefits, including the lowering of BP [153]. A recent study from our laboratory indicates that modulation of the gut microbiota by prebiotics or probiotics can prevent hypertension programmed by a perinatal high-fat diet [154]. Despite the demonstration in recent studies that microbiota-targeted therapies can be applied to several diseases [155], whether dietary amino acid supplementation can improve the gut microbiota and mucosal immunity, therefore benefiting the offspring’s BP, requires further exploration.

## 6. Conclusions

In pregnancy, amino acids play an essential role in fetal growth and development. This review highlights the impact of an imbalanced amino acid intake in pregnancy on the risk of developing hypertension in adult offspring. In particular, the targeting of specific amino acids as a reprogramming strategy opens a new avenue for preventing hypertension of developmental origin. However, this reprogramming strategy is in its infancy, so we must consider that any nutritional interventions in pregnancy could have unintended long-term consequences. So far, there remains a lack of accurate dietary recommendations for amino acid requirements for pregnant women. Given the complexity of amino acid metabolism between the mother and the fetus in pregnancy and the multifactorial nature of hypertension, elucidation of the pathophysiologic roles of specific amino acids and their interactions in the developmental programming of hypertension are needed before mothers and their children are able to benefit from using amino acid supplementation during pregnancy to prevent hypertension in adulthood.

## Figures and Tables

**Table 1:** Amino acid transporter systems in the human placenta.

Human Gene	Protein	System	Location	Substrate	Ref.
SLC1A1	EAAT3	X_AG_	MVM, BM	Anionic amino acids	[24,25]
SLC1A2	EAAT2	X_AG_	MVM, BM	Anionic amino acids	
SLC1A3	EAAT1	X_AG_	MVM, BM	Anionic amino acids	
SLC1A6	EAAT4	X_AG_	MVM, BM	Anionic amino acids	
SLC1A4	ASCT1	ASC	BM	Neutral amino acids	[25,26]
SLC1A5	ASCT2	ASC	BM	Neutral amino acids	
SLC3A1	rBAT	b^0,+^	?	Cationic and neutral amino acids	[27]
SLC3A2	4F2hc	L	MVM, BM	Neutral amino acids, BCAAs, and tryptophan	
SLC6A6	TAUT	β	MVM	Taurine	[28]
SLC7A1	CAT1	y^+^	MVM, BM	Cationic amino acids	[29]
SLC7A2	CAT2B	y^+^	MVM, BM	Cationic amino acids	
SLC7A3P	CAT3	y^+^	MVM, BM	Cationic amino acids	
SLC7A5	LAT1	L	MVM, BM	Cationic amino acids	[26,30]
SLC7A6	y^+^LAT2	y^+^L	MVM, BM	Cationic amino acids	
SLC7A7	y^+^LAT1	y^+^L	MVM, BM	Cationic amino acids	
SLC7A8	LAT2	L	MVM, BM	Cationic amino acids	
SLC7A10	ASC1	ASC	BM	Small neutral amino acids	
SLC7A11	xCT	X_c_^-^	?	Cysteine and glutamate	
SLC16A10	TAT1	T	BM	Aromatic amino acids	[26]
SLC38A1	SNAT1	A	MVM	Neutral amino acids	[31,32]
SLC38A2	SNAT2	A	MVM	Neutral amino acids	
SCL38A3	SNAT3	N	MVM	Neutral amino acids	
SLC38A4	SNAT4	A	MVM	Neutral amino acids	
SCL38A5	SNAT5	N	MVM	Neutral amino acids	
SLC43A1	LAT3	L	BM	Neutral amino acids	[26,33]
SLC43A2	LAT4	L	BM	Neutral amino acids	

SCL, solute carrier superfamily; MVM, microvillous membrane; BM, basal membrane; ?, unclear; BCAAs, branched chain amino acids. EAAT, excitatory amino acid transporter. ASCT, Alanine/Serine/Cysteine transporter. rBAT, related to b^0,+^ amino acid transporter. 4F2hc, 4F2 cell-surface antigen heavy chain. TAUT, taurine transporter. CAT, cationic amino acid transporter. LAT, large neutral amino acid transporter. ASC1, Alanine-Serine-Cysteine-1 transporter. xCT, the core subunit of the system X_c_^-^ high affinity cystine transporter. TAT1, T-type amino acid transporter. SNAT, neutral amino acid transporter. ASC, Alanine/Serine/Cysteine.

**Table 2:** Reprogramming interventions targeting amino acid supplementation to prevent the developmental programming of hypertension in rodent animal models.

Intervention	Animal Model	Species/Gender	Age at Measure	Ref.
Arginine/Taurine				
Arginine (20 g/L) and taurine (25 g/L) in drinking water plus antioxidants * from day 7 of gestation to postnatal week 4	Genetic hypertension	FHH/M and F	9 weeks	[88]
Arginine (20 g/L) and taurine (25 g/L) in drinking water plus antioxidants * from day 7 of gestation to postnatal week 8	Genetic hypertension	SHR/M and F	24 weeks	[89]
Arginine (20 g/L) and taurine (25 g/L) in drinking water plus antioxidants * from day 7 of gestation to postnatal week 8	Genetic hypertension	SHR/M and F	36 weeks	[90]
Arginine (20 g/L) and taurine (25 g/L) in drinking water plus antioxidants * from day 7 of gestation to postnatal week 4	Genetic hypertension	SHR/F	48 weeks	[91]
Taurine				
3% taurine in drinking water during pregnancy and lactation	High-sugar diet	SD/F	8 weeks	[92]
3% taurine in drinking water during pregnancy and lactation	Streptozotocin-induced diabetes	Wistar/M and F	16 weeks	[93]
3% taurine in drinking water during pregnancy and lactation	Genetic hypertension	SHR/M	22 weeks	[94]
5% taurine in drinking water during pregnancy	Genetic hypertension	SHRSP/M	3 months	[95]
Citrulline				
2.5 g/L citrulline in drinking water during pregnancy and lactation	Maternal 50% caloric restriction	SD/M	12 weeks	[96]
2.5 g/L citrulline in drinking water during pregnancy and lactation	Prenatal dexamethasone exposure	SD/M	12 weeks	[97]
2.5 g/L citrulline in drinking water during pregnancy and lactation	Streptozotocin-induced diabetes	SD/M	12 weeks	[98]
2.5 g/L citrulline in drinking water during pregnancy and lactation	Maternal L-NAME exposure	SD/M	12 weeks	[99,100]
2.5 g/L of water from day 7 of gestation to postnatal week 6	Genetic hypertension	SHR/M and F	50 weeks	[52]
Cysteine				
1% NAC in drinking water during pregnancy and lactation	Prenatal dexamethasone and postnatal high-fat diet	SD/M	12 weeks	[101]
1% NAC in drinking water during pregnancy and lactation	Suramin-induced pre-eclampsia	SD/M	12 weeks	[102]
1% NAC in drinking water during pregnancy and lactation	Maternal L-NAME exposure	SD/M	12 weeks	[103]
NAC (500 mg/kg/day) in drinking water from gestational day 4 to postnatal day 10	Maternal nicotine exposure	SD/M	8 months	[104]
Glycine				
3% glycine in chow during pregnancy and lactation	Maternal 9% protein restriction	Wistar/M	4 weeks	[105]
Branched chain amino acids				
BCAA-supplemented diets in pregnancy	Maternal 70% caloric restriction	SD/M	16 weeks	[106]

Studies tabulated according to type of amino acid, animal model, species, and age at measure. * Antioxidants: vitamin C (594 mg/L) in drinking water and vitamin E (9 g/kg) in chow. FHH, Fawn-hooded hypertensive rat; SD, Sprague–Dawley rat; SHR, spontaneously hypertensive rat; SHRSP, stroke-prone spontaneously hypertensive rat; M, male; F, female; L-NAME, N^G^-nitro–L-arginine methyl ester; NAC, N-acetylcysteine.

## Abbreviations

ADMA	Asymmetric dimethylarginine
AMPK	Cyclic adenosine monophosphate-activated protein kinase
BCAA	Branched chain amino acid
DOHaD	Developmental origins of health and disease
EAR	Estimated Average Requirement
FHH	Fawn-hooded hypertensive
GR	Glucocorticoid receptor
H_2_S	Hydrogen sulfide
IUGR	Intrauterine growth restriction
L-NAME	N^G^-nitro–L-arginine methyl ester
mTOR	The mechanistic target of rapamycin
NAC	N-acetylcysteine
NO	Nitric oxide
PPAR	Peroxisome proliferator-activated receptor
PGC-1α	PPARγ coactivator-1α
RDA	Recommended Dietary Allowance
SDMA	Symmetric dimethylarginine
SHR	Spontaneously hypertensive rat
SIRT	Silent information regulator transcript
